# From Gut to Goiter: How Salmonella Gastroenteritis Causes Thyroid Abscess

**DOI:** 10.1155/crie/2507383

**Published:** 2025-12-19

**Authors:** Andrew R. Cunningham, Hailey C. Lewis, Sean P. Holmes

**Affiliations:** ^1^ Brody School of Medicine, East Carolina University, Greenville, North Carolina, USA, ecu.edu; ^2^ Department of Otolaryngology, Eastern Carolina ENT, Greenville, North Carolina, USA; ^3^ Department of Otolaryngology, ECU Health Medical Center, Greenville, North Carolina, USA

**Keywords:** acute suppurative thyroiditis, salmonella abscess, thyroid abscess, thyrotoxicosis

## Abstract

**Introduction:**

The innate characteristics of the thyroid gland that resist infection render thyroid abscesses extremely uncommon. Moreover, gram‐negative species, such as *Salmonella* spp., are a rare cause of thyroid abscess from hematogenous dissemination.

**Case Description:**

A 41‐year‐old postpartum, breastfeeding female developed neck swelling and dysphagia following gastroenteritis. Imaging revealed a thyroid abscess. Fine needle aspiration (FNA) identified purulent fluid positive for *Salmonella* spp. Although the patient was initially responsive to antibiotics, recurrent swelling, dysphagia, and transient thyrotoxicosis developed, requiring re‐admission. Successful treatment included culture‐specific antimicrobials, ultrasound (US)‐guided needle drainage, and beta blockers, eliminating the need for surgical intervention.

**Conclusion:**

This case highlights a unique Salmonella thyroid abscess from hematogenous spread of infection in an immunocompetent individual. While thyroid abscesses, particularly those caused by *Salmonella* spp., are rare, clinicians should maintain a broad differential diagnosis, especially following gastrointestinal illness. FNA with culturing, imaging, and targeted antimicrobial therapy is crucial for timely diagnosis and favorable outcomes.

## 1. Introduction

Thyroid abscess formation is a rare entity, due to the gland’s vascularity, high iodine content, and encapsulation, which typically prevents infection [1]. Acute Suppurative Thyroiditis (AST) is seen when infection spreads to the thyroid gland, causing pain, fever, neck swelling, and possible abscess formation [2, 3]. AST represents less than 1% of all thyroid disease, with thyroid abscess being even rarer [2]. Risks increase in patients that have a history of Hashimoto’s thyroiditis, thyroid cancer, or are immunocompromised [4, 5].

While the majority of these cases are caused by gram‐positive cocci (notably *Staphylococcus aureus* and *Streptococcus pyogenes*), infections by gram‐negative bacteria are rare but increasingly recognized, particularly in at‐risk populations. *Salmonella* spp. represents an exceedingly rare etiology of gram‐negative infection in abscess formations. *Salmonella* spp. typically causes gastrointestinal disease, but hematogenous dissemination from an enteric source can potentially cause extraintestinal infections including an abscess. Salmonella‐associated thyroid abscesses are principally reported in adults with underlying immunodeficiency [2, 6–9] (e.g., diabetes mellitus, human immunodeficiency virus [HIV]/Acquired Immune Deficiency Syndrome [AIDS]), or pre‐existing thyroid pathology (nodules, goiter), with a minority occurring in children harboring congenital anomalies such as a pyriform sinus fistula [6].

Over the last several decades, there has been a paucity of reports describing Salmonella thyroid abscess, limited to only a few case reports and small case series specifically of immunocompromised patients. Here, we report an occurrence of this pathology in an immunocompetent patient with no risk factors.

## 2. Case Description

**Figure 1:**
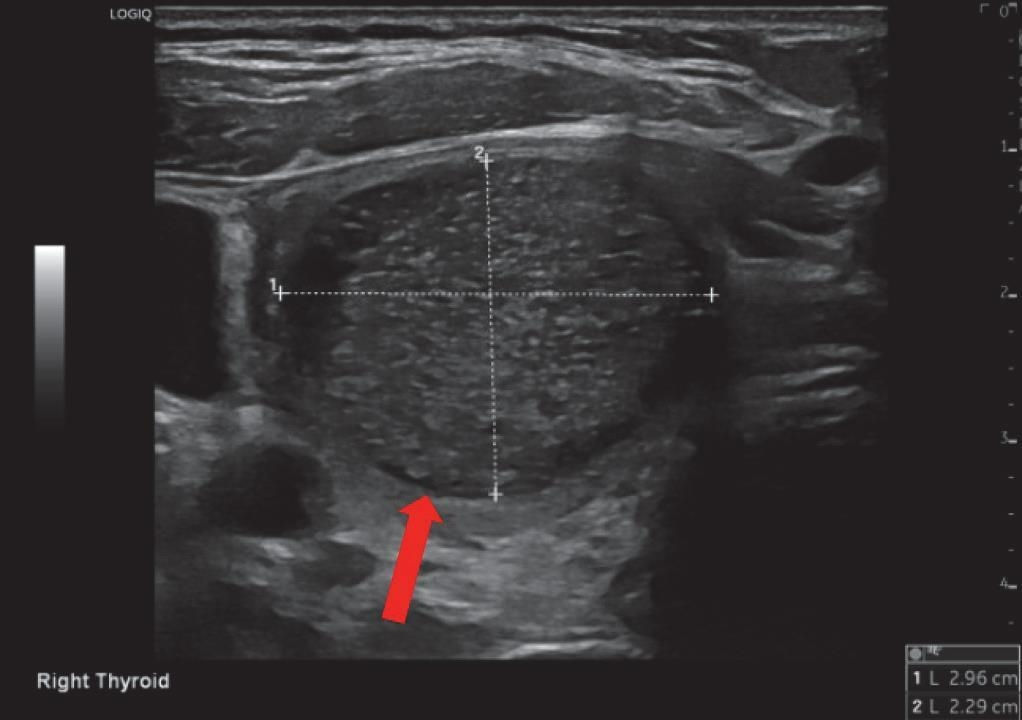
US right thyroid with red arrow demonstrating thyroid abscess formation.

**Figure 2:**
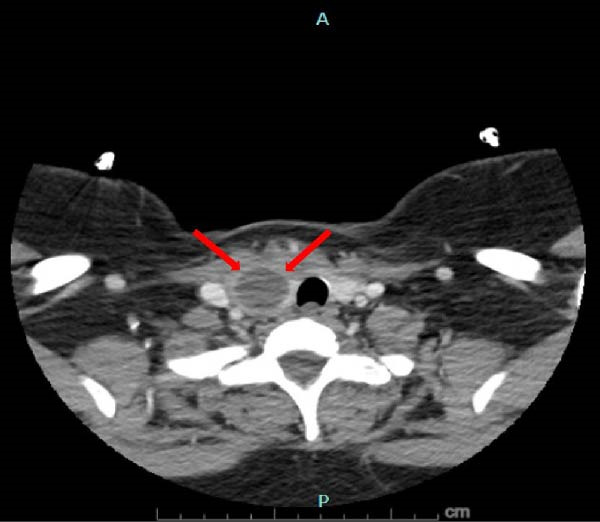
CT neck with red arrows demonstrating thyroid abscess formation.

A 41‐year‐old female with past medical history significant for recent childbirth, currently breastfeeding, with syncope and dizziness spells who initially presented to outpatient clinic with complaints of swelling and tenderness of the throat for 2 days. The pain was significantly worse when using straws and sneezing. She denied fever, chills, and sore throat. Recent history was significant for several days of diarrhea 1 week prior to presentation. There was no significant medical history of recurrent infections or skin conditions as would be seen in immunocompromised patients. The exam revealed a supple neck with tenderness along the anterior scalene muscles and neck swelling over the right thyroid. Ultrasound (US) thyroid showed a large hypoechoic lesion with tiny echogenic foci measuring 4.7 × 3.4 × 2.2 cm in the right lobe with suspected extrathyroidal extension (Figure 1). The left lobe and isthmus appeared normal. TI‐RADS 5. Lab values at this time showed a thyroid stimulating hormone (TSH) of 0.66 µIU/mL (Ref: 0.35–4.94 µIU/mL), normal comprehensive metabolic panel, with a complete blood count showing a white blood cell count of 12.5 k/uL (Ref: 4.5–11 k/uL) and absolute neutrophil number of 10.3 k/uL (Ref: 1.8–7.7 k/uL). Erythrocyte sedimentation rate was elevated to 51 mm/h (Ref: <20 mm/h) and C‐reactive protein elevated to 124.9 mg/L (Ref: <5 mg/L). Neck computed tomography (CT) showed a 5 cm predominantly cystic lesion in right thyroid with surrounding edema insinuating into retropharyngeal space (Figure 2) and was transferred to the hospital with concern for abscess 4 days after initial presentation to the clinic.

Initial hospital physical examination demonstrated worsening neck swelling with associated dysphagia, and fever to 103°F. Fine needle aspiration (FNA) was ordered due to concerning features, draining 17 cc of purulent brown fluid which had heavy growth of *Salmonella* spp. that was susceptible to all tested antibiotics (ciprofloxacin, ampicillin, trimeth‐sulfamethoxazole, and ceftriaxone). Patient was initially on IV ampicillin + sulbactam while awaiting cultures and was changed to IV ceftriaxone after cultures demonstrated susceptibility. Blood cultures showed no growths during hospitalization and patient demonstrated clinical improvement on antibiotics and was discharged on hospital day six with oral amoxicillin 1 g three times daily for 9 days to complete a total of 2 weeks antibiotics.

Hospital follow‐up 5 days later, the patient presented with complaints of worsening neck pain, swelling, and difficulties swallowing for the last 24 h. The patient was seen by otolaryngology clinic urgently after this visit due to concerns of the abscess refilling. At this appointment, otolaryngologist immediately referred the patient to the emergency department with concerns for thyroid storm due to palpitations, fever, sweating, thyromegaly, and tachycardia.

On admission, TSH became almost undetectable (0.01 µIU/mL) and free T4 was 2.99 ng/dL. During hospitalization, patient received 3 g ampicillin + sulbactam once, ceftriaxone 2 g IV for 5 doses inpatient and consultation from infectious disease recommended switching from amoxicillin to ciprofloxacin 500 mg oral twice daily upon discharge. Metoprolol was started for control of tachycardia and continued on discharge for tachycardia remaining >85 bpm. The patient was discharged and continued to report resolution of symptoms and decreasing size of thyroid abscess. Follow‐up testing after discharge for combined assay for HIV antibody and antigen was negative.

## 3. Discussion

Salmonella is a motile gram‐negative facultative anaerobic bacillus belonging to the *Enterobacteriaceae*. It is among the most common causes of foodborne illness globally, with an estimated 93.8 million cases yearly and more than 200,000 deaths [10–13]. Most causes are due to contaminated food products, including poultry, eggs, meats, produce, and unpasteurized milk [13, 14]. Those most at risk are children, elderly, immunocompromised, or individuals with sickle cell disease [13, 14]. Our patient was tested for HIV during one hospitalization to rule out immunocompromising conditions given Salmonella Abscesses higher prevalence in immunocompromised vs immunocompetent individuals. There are two main species, *Salmonella enterica* and *Salmonella bongori*, with enterica species being more common within humans [13]. Salmonella species are further divided into typhoidal salmonella (TS) including *S. typhi*, *S. paratyphi A*, *B*, *C* and nontyphoidal salmonella (NTS) including *S. enteritidis*, *S. typhimurium*, *S. newport*. Most reported thyroid abscesses involve NTS strains [13].

Though *Salmonella* spp. primarily targets the gastrointestinal tract, it can spread through hematogenous seeding or contiguous extension. In contiguous spread, likely due to congenital pyriform sinus fistula creates a tract from the hypopharynx to the thyroid gland [6, 15–17]. *Salmonella* spp. in the oropharynx from ingestion of contaminated products can spread via the fistula and is most common in children and young adults. Hematogenous seeding is more likely in adults, after initial gastrointestinal prodrome including fever, malaise, and an antecedent history of gastroenteritis 1–3 weeks before neck symptoms [13, 18]. *Salmonella* spp. invades the M cells in Peyer’s patches in the gastrointestinal symptoms, leading to bacteremia in 5–10% of cases [19]. Hematogenous seeding is most likely the cause in our patient with a history of gastroenteritis 1 week prior to neck pain, dysphagia, and neck mass. The associated dysphagia was most likely due to mass effect of the abscess rather than pyriform sinus infection, especially given the age of the patient with no previous neck symptoms or childhood infections.

Bacterial infection of the thyroid can cause AST, and rarely the formation of an abscess [3, 20]. Patients often have neck pain, fever, elevated white cell count, elevated ESR [20] as seen in our case. Inflammation can also result in hyperthyroidism, as damage to the thyroid follicles from mass effect or inflammation/infection can cause transient release of thyroxine (T4) and triiodothyronine (T3), suppressing upstream TSH from the anterior pituitary gland [3, 20]. This surge in released T3 and T4 causes thyrotoxicosis, resulting in the tachycardia, tremors, diaphoresis, and heat intolerance that sent our patient to the emergency room at the second hospitalization. The symptoms significantly overlap with hyperthyroid disorders, but thyrotoxicosis is transient in nature with therapy focused on symptomatic management and addressing the underlying cause (e.g., abscess, infection). Use of beta‐blockers in our case provided symptomatic management of the adrenergic symptoms and limiting the sympathetic nervous system response to excess thyroid hormone release [21–23]. Antibiotics should be tailored to cultures and susceptibility to treat underlying infectious cause.

Treatment for thyroid abscess varies by presentation, including both medical and surgical interventions. Antibiotics are crucial to treating the underlying cause of the abscess and thyroid infection. Drainage may be possible, using either incision and drainage, needle aspiration, or a percutaneous catheter [3, 24–27]. Severe cases with extensive neck infection may require partial or total thyroidectomy. The specific treatment is individualized based on the size, location, and stability of the patient. Our patient remained stable enough to only require IV antibiotics after initial FNA of the abscess with drainage of 17 cc purulent fluids.

This case highlights the importance of maintaining a broad differential diagnosis when evaluating anterior neck swelling, particularly in patients with recent systemic symptoms such as gastroenteritis. Although thyroid abscesses are rare due to the gland’s natural resistance to infection, clinicians should remain vigilant for atypical pathogens like *Salmonella* spp. Hematogenous spread following gastrointestinal infection can lead to thyroid involvement even in immunocompetent patients, as seen in this postpartum woman. Key clinical signs—including localized neck tenderness, elevated inflammatory markers, and thyrotoxic symptoms—should prompt early imaging and consideration of infectious thyroiditis. Transient thyrotoxicosis can mimic hyperthyroid disorders but is managed symptomatically with beta‐blockers rather than antithyroid drugs (e.g., methimazole, propylthiouracil). FNA with culture remains essential for diagnosis and targeted antimicrobial therapy. Prompt recognition, appropriate antibiotic coverage, and drainage when indicated can prevent complications and ensure favorable outcomes. Early ENT consultation is advised in cases of recurrent or worsening symptoms, in addition to endocrinology and infectious disease as indicated.

## 4. Conclusion

This case underscores the importance of recognizing *Salmonella* spp. as a rare cause of thyroid abscess. A history of recent gastroenteritis should raise suspicion for hematogenous spread of *Salmonella* spp. Transient thyrotoxicosis secondary to glandular inflammation may mimic hyperthyroid states and should be managed supportively with beta‐blockers and targeted antibiotic therapy if signs of bacterial infection are present. Early imaging, microbiologic diagnosis, multidisciplinary care, and appropriate drainage are all crucial for effective treatment and recovery. Awareness of this rare presentation can aid clinicians in timely intervention, reducing morbidity associated with delayed diagnosis or treatment failure.

## Consent

No written consent has been obtained from the patients as there is no patient identifiable data included in this case report.

## Disclosure

IRB approval was not required for case reports, and no identifying information was included in this report.

## Conflicts of Interest

The authors declare no conflicts of interest.

## Author Contributions

**Andrew R. Cunningham**: data curation, formal analysis, investigation, methodology, project administration, supervision, visualization, writing – original draft preparation, writing – reviewing and editing. **Hailey C. Lewis**: investigation, data curation, writing – reviewing and editing. **Sean P. Holmes**: conceptualization, data curation, resources, writing – reviewing and editing.

## Funding

The authors received no specific funding for this work.

## Data Availability

The data that support the findings of this study are available on request from the corresponding author. The data is not publicly available due to privacy or ethical restrictions to protect patient privacy.
